# Effect of Green Food Processing Technology on the Enzyme Activity in Spelt Flour

**DOI:** 10.3390/foods11233832

**Published:** 2022-11-27

**Authors:** Maja Leitgeb, Željko Knez, Gordana Hojnik Podrepšek

**Affiliations:** 1 Laboratory for Separation Processes and Product Design, Faculty of Chemistry and Chemical Engineering, University of Maribor, Smetanova ulica 17, SI-2000 Maribor, Slovenia; 2 Faculty of Medicine, University of Maribor, Taborska ulica 8, SI-2000 Maribor, Slovenia

**Keywords:** food treatment, spelt flour, supercritical carbon dioxide, enzyme inactivation, defatted flour, carbon dioxide solubility

## Abstract

In this research, a new approach to enzyme inactivation in flour was presented by supercritical technology, considered a sustainable technology with lower energy consumption compared to other technologies that use ultra-high temperature processing. Total protein concentration and the activity of enzymes α-amylase, lipase, peroxidase, polyphenol oxidase, and protease were determined in flour pre-treated with scCO_2_. During the study, it was observed that the activity of enzymes such as lipase and polyphenol oxidase, was significantly reduced under certain conditions of scCO_2_ treatment, while the enzymes α-amylase and protease show better stability. In particular, polyphenol oxidase was effectively inactivated below the 60% of preserved activity at 200 bar and 3 h, whereas α-amylase under the same conditions retained its activity. Additionally, the moisture content of the scCO_2_-treated spelt flour was reduced by 5%, and the fat content was reduced by 58%, while the quality of scCO_2_-treated flour was maintained. In this regard, the sustainable scCO_2_ process could be a valuable tool for controlling the enzymatic activity of spelt flour since the use of scCO_2_ technology has a positive effect on the quality of flour, which was verified by the baking performance of spelt flour with the baked spelt bread as an indicator of quality.

## 1. Introduction

The food industry is currently facing several challenges, where the biggest concern is the consumer’s decision to select food for consumption. Today’s technologies are mainly aimed at providing the consumer with quality food, as minimally processed as possible, but, at the same time, with the possibility of maintaining the freshness of food for a long time. Therefore, researchers have recently been focusing on research that would achieve higher food quality and extend the shelf life of a particular food [1]. For several decades, various researchers have been discovering the use of high-pressure technology on food quality, especially on its nutrients. Much research has been conducted to pasteurize food with high-pressure technologies, mostly high hydrostatic pressure [2,3]. Moreover, due to the influence of enzymes that catalyze specific reactions in certain foods, the function of enzymes in food is becoming increasingly important. It is known that certain industries use the addition of specific enzymes for faster production with better products. In this way, they affect the economy of a particular product’s production process and the quality. Despite the fact that a lot of research has been conducted on different fruit and vegetable juices using high-pressure technologies, only a handful of papers can be found in the literature that write about the activity of enzymes in flour [4,5,6]. In the baking industry, hydrolases, such as amylases, proteases, and lipases, are commonly used. These enzymes act on polymers (starch, proteins, and lipids), and they require water for their operation [7]. For this reason, our research focuses on identifying enzymes found in flour, their activity, and the positive and negative effects triggered by specific enzymes. In this research paper, the enzymes found in spelt flour are precisely defined, with great emphasis on their activity, with a particular focus on their activity after processing with scCO_2_. It is well known that supercritical fluids are substances above their critical temperature and pressure [8]. Their important advantage is that their physical and chemical properties are between those of the liquids and gases. Therefore, they predominate over other thermodynamic phases (solid, liquid, and gaseous). In addition, their properties may be very easy to adjust simply with minor changes in pressure or/and temperature. Supercritical fluids have a similar density as liquids and viscosity near gases. In combination with the low surface tension, their physical properties provide rapid penetration into cells or particles. Due to the transport properties of supercritical fluids, they may be used in various applications, including enzyme inactivation [9]. High-pressure technology using CO_2_ under supercritical conditions is an approach that offers many suitable properties that are crucial in food processing [10]. In addition, a lot of CO_2_ is available with a relatively low price, and it is approved as a solvent in the food industry [11]. The advantage of this method is in the fact that CO_2_ is not considered as an expensive gas and process temperatures are particularly low. Heat consumption is not required, which is important from an economic and ecological point of view. Certainly, the most expensive part is the process equipment for scCO_2_ technology. However, the most important fact is an important and positive environmental impact due to the high-pressure processing implementation, as a food production process is achieved because of substantial water and energy savings. The use of sustainable scCO_2_ technology, where CO_2_ is considered as a green solvent, provides processes which attract considerable interest. In this paper, some relevant advantages of using scCO_2_ to improve the quality of flour and, consequently, bakery products are presented. Based on these facts, it is highly recommended from an environmental, safety, and economical point of view to develop alternative routes for reducing the activity of undesirable enzymes. Furthermore, due to the extension of the shelf-life of processed products, this allows a significant reduction of food waste [12]. On the other hand, it is important to emphasize that a system that would enable scCO_2_ treatment is an expensive investment, but the costs of serial production are expected to be lower, especially for CO_2_, which is considered a cheap gas.

The supercritical phase of CO_2_ can be achieved at relatively low pressure and temperature (7.38 MPa and 31.2 °C) [13], so as not to damage biological substances significantly. In many research studies, as well as industries, scCO_2_ is the most widely used supercritical fluid, with the very important possibility of achieving a non-thermal (35–55 °C) process. In relation to that, our research focused on enzyme inactivation in spelt flour, which was exposed to various scCO_2_ conditions. Additionally, the effects of the non-thermal scCO_2_ processing of the spelt flour on the nutritional compounds, physicochemical properties, sensory attributes, and bread quality, as well as its enzymatic inactivation, were discussed. 

In the beginning, it is crucial to understand the importance of specific enzymes and their role in the food production. Therefore, the results of enzyme inactivation by scCO_2_ in certain foods are summarized in Table 1. From Table 1, it is evident that much research was focused on the inactivation of the two very important enzymes in food, polyphenol oxidase (PPO) and peroxidase (POD). The reason is that these two enzymes cause many adverse effects on food storage stability, especially in fresh foods, such as fruits and vegetable juices. These two enzymes are also crucial in flour, as they cause a browning reaction and accelerate oxidation, which shorten a product’s shelf life. 

Furthermore, with the use of scCO_2_ technology for inactivation of enzymes in fruit and vegetables, enzymatic browning and off-flavor formation is prevented. These changes lead to degradation and quality changes of juices during storage. Enzymatic browning is a major problem in the beverage industry (apple juice), as PPO oxidizes the o-diphenols to quinones, leading to polymerization, which produces brown pigment (melanin) [14]. Our research also focuses on the inactivation of PPO and POD, whereby certain enzymes such as α-amylase, protease, and lipase were also detected, since these enzymes have a significant impact on the bread baking process.

**Table 1:** Examples of food enzyme inactivation using scCO_2_.

Inactivated Enzyme	Process Conditions	Residual Activity [%]	Processing Media	Reference
**Alkaline phosphatase (ALP)**	70 °C, 8 MPa, 30 min	6	Milk	[15]
**α-amylase**	35 °C, 30 MPa, 24 h	40	α-amylase from *A. oryzae*	[16]
**Cellulase**	41 °C, 12 MPa, 150 min	48	Cellulase enzyme from *Trichoderma* *longibrachiatum*	[17]
**Lipase Lipozyme 435**	60 °C, 20 MPa, exposure time (6 h) and depressurization steps (1–3)	30	Lipase from *Candida antarctica* (Lipozyme 435, food grade) immobilized on a macroporous anionic resin	[18]
**Lipoxygenase (LOX) Peroxidase (POD)**	50 °C, 10.3 MPa, 15 min	1	Lipoxygenase from soybean	[19]
	55 °C, 62.1 MPa, 15 min	10	Peroxidase from horseradish	
**Polyphenol oxidase (PPO)**	55 °C, 25 MPa, 20 min	0	Apple juice	[20]
**Polyphenol oxidase (PPO)** **Peroxidase (POD)** **Pectin esterase (PE) Polygalacturonase (PG)**	39 °C, 10 MPa, 30 min	90	Beetroot juice	[21]
	31 °C, 60 MPa, 30 min	94		
	39 °C, 10 MPa, 30 min	92		
	39 °C, 10 MPa, 30 min	93		
**Polyphenol oxidase (PPO)**	55 °C, 12 MPa, 15 min	0	Apples in syrup	[22]
**Polyphenol oxidase (PPO)**	55 °C, 30 MPa, 60 min	40	Cloudy apple juice	[23]
**Polyphenol oxidase (PPO)**	37 °C, 25MPa, 10 min	10	Pacific white shrimp	[24]
**Polyphenol oxidase (PPO)**	40 °C, 10 MPa, 20 min	3	Apple juice	[20]
**Polyphenol oxidase (PPO)** **Horseradish peroxidase (POD)**	50 °C, 65 MPa, 30 min	12 1	Mushroom polyphenol oxidase from *Agaricus bisporus*; Horseradish peroxidase from *Amoracia rusticana* roots	[25]
**Polyphenol oxidase (PPO) Peroxidase (POD)**	35 °C, 60 MPa, 30 min	0 65	Strawberry juice	[26]
**Polyphenol oxidase (PPO) Peroxidase (POD)**	45 °C, 60 MPa, 30 min	20 47	Cloudy apple juice	[27]
**Polyphenol oxidase (PPO) Peroxidase (POD)**	50 °C, 25 MPa, 6 h	34 0	Mate tea leaves	[28]

The temperatures during scCO_2_ processing are usually below 50 °C. ScCO_2_ technology works with pressures between 8 and 60 MPa [9]. Zhang et al. processed Pacific white shrimp, using scCO_2_ technology for the inactivation of polyphenol oxidase (PPO). The optimal process conditions were obtained at 37 °C, 25 MPa, and 10 min, which are very similar to the conditions used in this study. Moreover, only 10% of PPO residual activity was maintained [24]. Murtaza et al. and Ferrentino and Spilimbergo evaluated the scCO_2_ processing of apple juice, aiming to inactivate PPO, both achieving total PPO inactivation, but Ferrentino and Spilimbergo used lower pressure conditions and shorter treatment time, which are more economical [20,22]. In addition to a successful and satisfactory result, the economic aspect is also essential, especially for applications included in food production. Indeed, interesting is the result of a study of beetroot juice from Marszałek, which suggests that too low of a pressure was probably used, as the inactivation of various enzymes, such as PPO, POD, pectin esterase, and polygalacturonase has not been as successful [21]. The cause for such inefficient enzyme inactivation may also be the result of the food composition, as it is crucial where the enzymes are located in food and how they are accessible to CO_2_. 

Our study included research on the influence of different scCO_2_ conditions on the activity of specific enzymes in spelt flour. Obtained results were an important accomplishment for the further use of spelt flour in the production process. However, it must be known what reactions are catalyzed by specific enzymes in spelt flour and why additives in the form of enzymes are increasingly being used in the bakery industry, ultimately contributing to improved production. Previously published studies have focused mainly on the addition of certain enzymes to flour and the consequent monitoring of the rheological characteristics of the dough by the addition of different enzyme concentrations. In our research, the emphasis was on already existing enzymes in spelt flour, which, with the green approach using scCO_2_ technology, improve or reduce the enzyme activity according to the individual type of enzyme. Therefore, the novelty of this study was the approach by which the inactivation of enzymes in flour occurs by the use of scCO_2_ technology. So far, there has been a greater emphasis on maintaining quality in preserving fruit and vegetables and inhibiting the growth and reproduction of microorganisms [29]. Upgrading this, our study defines the beginnings of the use of scCO_2_ technology in flour, where the importance of enzymes has not been well researched so far. It is of the utmost importance that this process does not negatively affect the quality of flour as a raw material for bakery products. It is definitely necessary to have a good knowledge about the enzymes present in the flour and their function, especially in the production process of the final product and its quality.

## 2. Materials and Methods

### 2.1. Materials

Spelt flour used in this study was obtained by grinding the cleaned whole grain of spelt wheat (*Triticum spelta*) from the company Hlebček d.o.o., Pragersko, Slovenia. Ethanol (96%), phosphoric acid (≥85%), sodium chloride (≥99.5%), Coomassie brilliant blue G250 (1.15444.0025), and acetonitrile (99.9%) were supplied from Merck. Chicken egg albumin (≥98%), sodium acetate (≥99.0%), acetic acid (GR for analysis), and p-nitrophenyl butyrate (≥98%) were supplied from Sigma. The enzymes α-amylase (~30 U/mg) from *Aspergillus oryzae* and protease (≥0.6 U/mg) from *Aspergillus saitoi* were obtained from Sigma, while lipase (~200 U/g) from *Aspergillus niger* was obtained from BioChemics. Peroxidase (232-668-6) from horseradish was purchased from BBI Enzymes (Blaenavon, UK). Polyphenol oxidase (LS003793) from *Agaricus bisporus* was supplied from Worthington Biochemical Corporation. All the chemicals were used as received without any further purification. All other chemicals used in the laboratory were of analytical grade.

### 2.2. Methods

#### 2.2.1. Equipment and Processing Protocols

**Figure 1:**
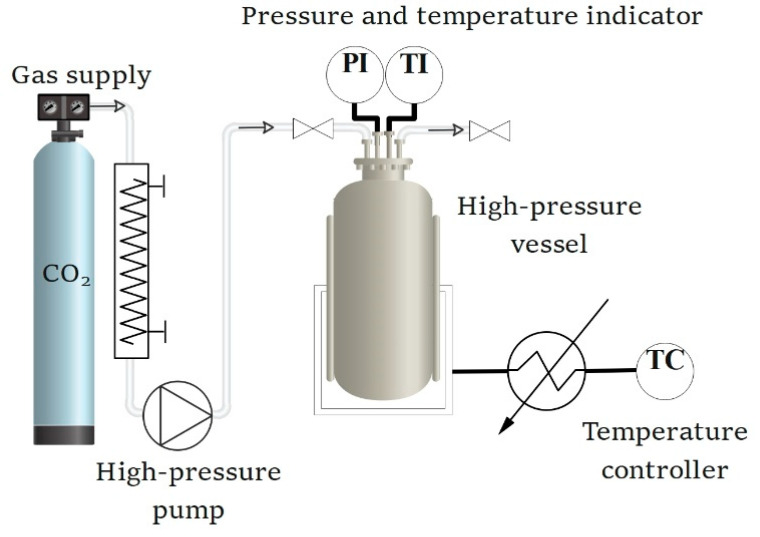
Schematic illustration of the experimental scCO_2_ high-pressure reactor.

The experimental system is schematically illustrated in Figure 1. A scCO_2_ system is comprised of a 60 mL high-pressure vessel fitted with the appropriate addition modules, heating, temperature control, safety features, flow meters, and pressure controls valves.

In a typical experiment, 5 g of spelt flour was weighed into a filter bag, which was then placed into the pressure vessel, where the temperature was maintained at 35 °C. A treatment time was defined as that after the system reached the set pressure and temperature. CO_2_ (99.5% purity, Messer, Ruše, Slovenia) from a cylinder was cooled to 3 °C by a heat exchanger and delivered by a CO_2_ pump. After treatment with CO_2_, the vessel was slowly depressurized, and the sample was collected and immediately used for further protein extract preparation. Treatments were performed in triplicate.

#### 2.2.2. Protein Extract Preparation

Proteins were extracted from spelt flour in accordance with the established procedure [30], where 5 g of flour was suspended in 30 mL 0.1 M acetate buffer (pH 5.3), and the suspension was shaken for 1.5 h at room temperature, followed by centrifugation at 8000 rpm for 5 min. The obtained supernatant was an extract of flour proteins.

#### 2.2.3. Determination of Protein Concentration

The protein concentration was determined directly by measuring protein content in the extract of flour proteins with UV-spectrophotometer using the Bradford dye-binding method [31]. Moreover, bovine serum albumin (BSA) was used as the protein standard to obtain a standard curve ranging from 0 to 1 mg/mL BSA.

#### 2.2.4. Measurement of Specific Enzyme’s Activity

The activity of a specific enzyme was determined based on specific enzymatic activity assays using a UV-spectrophotometer (Varian, Cary 50 Probe, Agilent Technologies, Santa Clara, CA, USA) at different wavelengths.

##### α-Amylase Activity Assay

The α-amylase activity was determined based on the increased glucose in the solution using the DNS reagent (3,5-dinitrosalicylic acid). 1 g DNS was stirred with 20 mL of 2 M sodium hydroxide, and the mixture was diluted with 50 mL water. Afterwards, 30 g of sodium potassium tartrate was added and dissolved. The incubation mixture contained enzyme extract (0.5 mL) and 1% substrate (starch from wheat) in 0.02 M sodium phosphate buffer (0.5 mL). The incubation mixture was mixed with 1 mL DNS reagent and kept in a boiling water bath for 5 min. After cooling the incubated tubes, 10 mL reagent grade water was added. The absorbance was measured with a UV-Vis spectrophotometer at a wavelength of 540 nm.

##### Lipase Activity Assay

Briefly, an incubation mixture was prepared by the addition of a solution of 100 mM sodium phosphate buffer with 150 mM sodium chloride and 0.5% (*v*/*v*) Triton by adding 0.10 mL of enzyme extract from spelt flour. The contents were mixed, and the tubes were incubated in the water bath at 37 °C for 3 min. Then, 10 µL of substrate solution (50 mM p-nitrophenyl butyrate (pNPB) diluted in acetonitrile and stored at −20 °C) was added. The activity of lipase was determined based on p-nitrophenol (pNP) reaction, which was monitored at 400 nm for 5 min. One unit of lipase activity released 1 nanomole of pNP per minute at pH 7.2 and 37 °C, using p-nitrophenyl butyrate (pNPB) as the substrate. 

##### Peroxidase Activity Assay

Peroxidase activity was measured using phenol, 4-aminoantipyrine (4-AAP), and hydrogen peroxide as substrates. Our previous research has published the method for peroxidase activity determination and detailed calculation procedure of peroxidase activity [32].

##### Polyphenol Oxidase Activity Assay

PPO activity was determined spectrophotometrically at 265 nm by monitoring the reaction at 25 °C for 5 min. The final concentrations in the reaction mixture were 50 mM potassium phosphate, 0.17 mM L-3,4-dihydroxyphenylalanine, 0.072 mM ascorbic acid, 0.0022 mM ethylenediaminetetraacetic acid, and 50–100 units of PPO. One unit of enzyme activity was defined as the amount of enzyme causing a 0.001 change in the absorbency per minute at pH 6.5 at 25 °C in a 3 mL reaction mix [33].

##### Protease Activity Assay

Protease activity was measured using casein as a substrate. A procedure for determining the protease activity was previously described in our research [34].

All experiments were conducted in triplicate, and the error bar represents the percentage error (±3%) in each set of readings.

##### Statistical Analysis

All determinations were conducted at least in triplicates. Analysis of variance (ANOVA) and Duncan’s multiple range test were employed to determine the statistical significance of the differences between the means (*p* ≤ 0.05) by using IBM SPSS Amos 28.

#### 2.2.5. Flour Characterization Methods

Scanning electron microscopy (SEM), attenuated total reflectance—Fourier transform infrared spectroscopy (ATR-FTIR), moisture content, and fat content were carried out for characterization of untreated spelt flour and scCO_2_-treated spelt flour.

A scanning electron microscope (SEM) model Quanta FEI 200 3D microscope (FEI, Hillsboro, OR, USA) was used to study the microstructure of the sample. The pressure chamber was 60 Pa, and the accelerating voltage for imaging was 10 kV. 

ATR-FTIR (Shimadzu, IRAffinity-1s) was used for functional group identification of untreated and scCO_2_-treated spelt flour. To test repeatability, analyses were performed in triplicate and average spectra were used.

The moisture content in untreated spelt flour and spelt flour that was exposed to scCO_2_ at a pressure of 300 bar and 35 °C in a high-pressure batch reactor for 24 h was determined using the Mettler Toledo HX204 Moisture Analyzer halogen moisture analyzer with a heating temperature of up to 130 °C.

Soxhlet apparatus was used to extract fats from untreated and SCCO_2_-treated spelt flour samples with petroleum ether as a solvent, according to Official Method AOAC 963.15, with minor modifications [35]. After the extraction, the solvent was evaporated from the extraction mixture using rotavapor, the mass of the extract obtained was determined, and the fat extraction yield was calculated. Three replicates were made for each sample.

Equilibria solubility of CO_2_ was studied by the volumetric expansion of spelt flour saturated with CO_2_ on a magnetic suspension balance (MSB). The solubility of CO_2_ is the total amount of carbonate species that can be dissolved in the solution. Solubility of CO_2_ in spelt flour was measured by a gravimetric method involving magnetic suspension balance (MSB, RUBOTHERM, Bochum, Germany) at pressures ranging from 1 bar to 300 bar. The temperature of the cell was kept constant using a Lauda P5 thermostat. The software MessPro recorded the conditions (mass, temperature, pressure). The duration of the measurement at each pressure was approximately 120 min, as after this time, equilibrium was reached. To define the volume of the sample, a photo of the sample was taken with a digital camera and analyzed using ImageJ software [36].

#### 2.2.6. Characterization of Baked Bread

The specific volume of baked bread was determined by the water displacement method. Loaf weight was recorded, and specific volume (cm^3^/g) was calculated. Ten readings were taken from baked bread to determine the specific volume. Meanwhile, loaf height and crumb thickness were measured using ImageJ software, where loaves were vertically sliced in the centre.

## 3. Results and Discussion

### 3.1. Influence of scCO_2_ Process upon Enzyme Activity Loss

**Figure 2:**
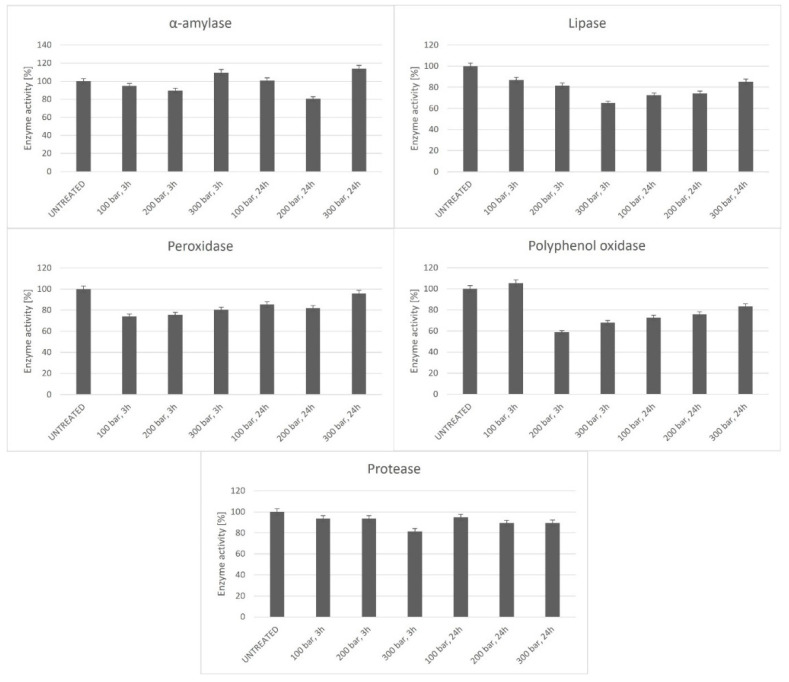
The effect of scCO_2_ on enzyme activity in spelt flour. Data represent mean ± SD of three independent experiments. Means among each set of data labelled by the exposure conditions were not significantly different (*p* < 0.05) by Duncan’s multiple range test.

In this study, a green approach using scCO_2_ technology was used to process spelt flour. ScCO_2_ technology was checked for its great advantages for food processing; this involved its ability to preserve food quality due to the mild process conditions and, at the same time, to achieve a change in the activity of specific enzymes. The activities of α-amylase, lipase, peroxidase, polyphenol oxidase, and protease, which were detected in spelt flour before and after scCO_2_ treatment at different parameters, are presented in Figure 2. Data show that scCO_2_ affected the activity of individual enzymes, depending on the type of enzyme and process parameters. The most favorable parameters for α-amylase enzyme inactivation were 200 bar and 24 h, with 20% enzyme inactivation. The lipase and protease inactivation results showed that the highest level of inactivation was achieved at 300 bar and 3 h of exposure, as lipase activity achieved 65% under these conditions and protease achieved 81%. A minimum POD activity of 74% was observed at process exposure conditions of 100 bar and 3 h, whereas PPO activity was the lowest (59%) at 200 bar and 3 h. Oxidation of amino acids present in free and polypeptide chains is a primary mechanism of enzyme inactivation in scCO_2_, with additional occurrences of depolymerization of polypeptide chains and destruction of secondary structural elements of enzymes [37].

Pressures of 100, 200, and 300 bar were used in the experiments. The data showed that it was not always the highest pressure that caused the highest enzyme inactivation. An interesting finding was that a higher level of enzyme inactivation was achieved even with a shorter exposure time (3 h). It was shown that the α-amylase activity of flour directly affected the fermentability of flour and the quality of fermented foods, therefore the process parameters, 300 bar and 3 h, were set in continuation of the experiment, which, in most cases, represented the conditions where the highest level of inactivation of enzymes that negatively affect the further process of preparation of the bakery product was achieved.

In many studies, CO_2_ treatment was more effective in the inactivation of various enzymes with the prolongation of the exposure time. This technology physicochemically inactivates enzymes at a relatively low temperature (≤60 °C) and moderate pressures (8–40 MPa) without residual waste generation [38]. However, inactivation increase is not always achieved with the prolongation of the exposure time. Fleury et al. found, similar to our study, that the increase in inactivation with time was possible only until the treatment period reached certain parameters, and beyond these there was no further improvement, even when the treatment was prolonged [39]. Only the pressure increase may cause higher inactivation. This fact depends on the enzyme, as confirmed in this study. Higher pressure can increase CO_2_ solubilization, facilitating its contact with and penetration into food. This is the reason for higher enzyme inactivation efficiency. At the same time, higher pressure can generate the same inactivation efficiency at lower temperatures. Despite that, higher pressure can induce a decrease in pH, leading to enzyme inactivation [10]. The direct effect of scCO_2_ on enzyme inactivation is the formation of carbamates with lysine residues on the enzyme surface. In addition, carbonic acid is formed and pH decreases [40].

If amylases are applied the staling rate of bread, this may result in reduction, which results in the most successful enzymatic approach. Amylases catalyze the cleavage of 1,4-glycosidic bonds. The starch molecules are, therefore, degraded. Dextrin with lower molecular weight and other short-chain unbranched molecules are formed. Additionally, amylases are the reason for a greater loaf volume, improved crumb structure, and, at the same time, starch retrogradation is hindered [41]. Therefore, it is essential to maintain or increase the amylase activity in flour, as this contributes to a better final product, which, from our results using scCO_2_ technology, was also achieved. In the process of scCO_2_, PPO in the spelt flour was inactivated to prevent the enzymatic oxidation of spelt flour. Generally, quinones in food are produced because of the reaction of naturally present phenolic compounds with PPO enzymes. In the case of flour, this can lead to the darkening of flour dough. For this reason, it was proven that the use of scCO_2_ successfully inactivated the enzyme PPO and thus reduced the occurrence of enzymatic browning reactions in spelt flour. As shown, the highest inactivation of PPO was reached at 200 bar and 3 h, whereas the inactivation of amylase at these conditions was around 10%.

On the other hand, some flavor compounds are produced by lipid degradation and oxidation pathways. Therefore, understanding lipid degradation and oxidation pathways are important in the quality control of spelt flour and products. Lipase catalyzes the first step of lipid catabolism, which can cause deterioration of food quality [42]. Moreover, POD activity plays an essential role in flour-based product quality, which is mainly associated with flour’s browning and bleaching effects, since POD belongs to the class of oxidoreductases and performs a catalytic function in the oxidation of various organic and inorganic substances with the participation of hydrogen peroxide [43]. This indicates that a lower content of lipase and POD enzymes is desirable in the flour. As depicted in Figure 2, the enzymes POD and lipase were also successfully inactivated using scCO_2_, which would effectively extend the shelf-life of spelt flour. 

To clarify the effects of enzymes in flour, which were previously scCO_2_-treated, on quality deterioration, characteristics of spelt flour and sensory analysis of a baked product, untreated and scCO_2_-treated spelt flour analyses were performed and evaluated below.

### 3.2. SEM and FTIR Characteristics of Spelt Flour

**Figure 3:**
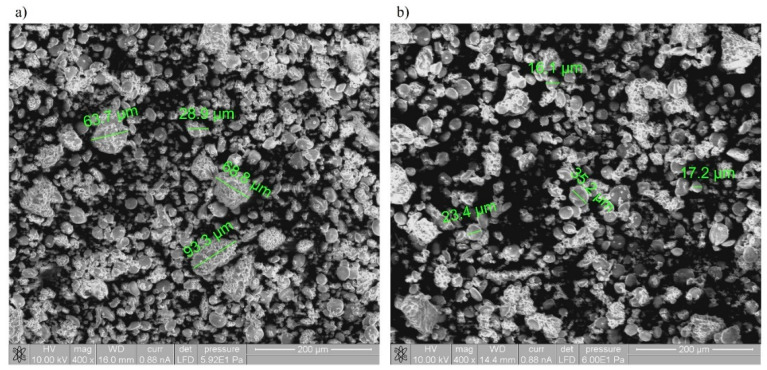
Scanning electron micrographs (SEM) of (**a**) untreated spelt flour and (**b**) scCO_2_-treated spelt flour. Magnification: ×400.

The structural properties of untreated and scCO_2_-treated spelt flour were characterized using SEM analysis. The differences in microstructure of untreated and scCO_2_-treated spelt flour were obtained to contribute to the knowledge about the consequences of the use of scCO_2_-treated spelt flour in the bakery. In order to assess the role of scCO_2_ on the structure of starch grains of spelt flour, SEM and FTIR analyses were performed to confirm the differences between spelt flour before and after exposure to scCO_2_. The SEM images of spelt flour samples obtained before and after scCO_2_ treatment are shown in Figure 3. Initially, the SEM image showed clearly visible smooth starch granules, which did not appear to be damaged, even after scCO_2_ treatment. The starch granule size of spelt flour was determined in the range of 16–35 µm. Certainly, the SEM analysis clearly showed the difference in the formation of aggregates in the size of 100 µm in untreated spelt flour, which can be explained by the fact that the moisture content in untreated spelt flour was minimally higher (4.7%) than in scCO_2_-treated spelt flour because of carbonic acid formation. Thus, these results were in accordance with the moisture and fat content measurements. In addition, it was observed that the scCO_2_-treated flour was more free-flowing, as it also contained less fat. SEM analysis confirmed that scCO_2_ did not adversely affect flour quality, and, in addition, changes in flour properties such as reduced fat and moisture content in scCO_2_-treated spelt flour were observed.

**Figure 4:**
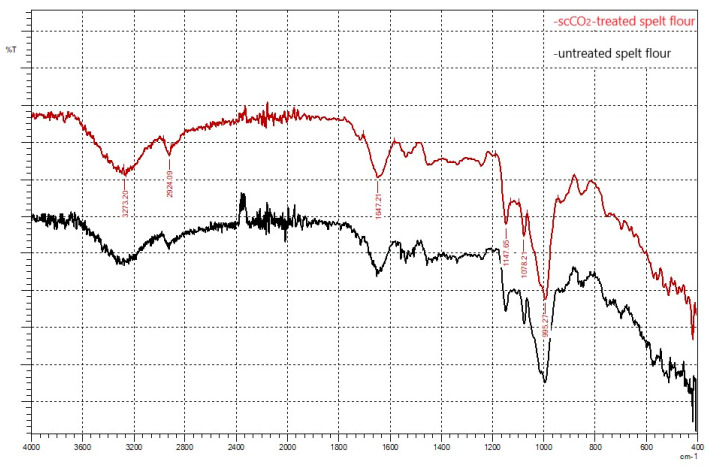
FTIR patterns of the untreated spelt flour (black) and scCO_2_-treated spelt flour (red).

A FTIR spectrum of untreated and scCO_2_-treated spelt flour shows the contribution of different functional groups belonging to carbohydrates, lipids, proteins, and others, according to the main chemical composition of flour. FTIR analysis was performed for untreated and scCO_2_-treated spelt flour using an ATR accessory. Samples’ spectra were obtained over the range of 400 cm^−1^ and 4000 cm^−1^ with a spectral resolution of 0.5 cm^−1^. Figure 4 presents the FTIR analysis used for qualitative observation of changes in functional groups and further assessment of component modification. Figure 4 showed that there was no significant change in the FTIR pattern of untreated and scCO_2_-treated spelt flour, as the characteristic peaks occur at the same values in both spectra. The results demonstrated the band ~1078 cm^−1^ was related to the crystalline structure of starch, and the absorption band at ~995cm^−1^ in the starch region was linked to hydrated crystalline domains. A similar observation was reported previously by Zhou et al. in potato starch [44]. Moreover, FTIR patterns promptly indicated the presence of starch (1200–800 cm^−1^), lipids (2924, 2854, and 1740 cm^−1^), and proteins (1650–1500 cm^−1^) in both spelt flour samples, where minor differences, quantitative or structural, were difficult to detect [45].

Taken together, these data demonstrate that there was no change in the intensity of the characteristic peaks of FTIR spectrum, which means that scCO_2_ does not affect major functional groups of spelt flour.

Solaesa et al. reported structural, physicochemical, pasting, and thermal properties of quinoa defatted by scCO_2_ extraction, where it was confirmed by SEM that native full-fatted quinoa starch granules were presented as aggregations. Comparable to the results of this study, it was found that scCO_2_ defatted quinoa samples was more disordered, presenting less aggregation between the components [46], as was also the case according to our SEM analysis of scCO_2_-treated spelt flour-less aggregates, and greater lightness of the sample were detected.

In comparison, Ma et al. treated wheat flour with superheated steam, where partial gelatinization of starch granules and protein denaturation induced starch–starch and starch–protein aggregate formation, which changed the particle size distribution and resulted in the increase in flour particle size. Specifically, the FTIR spectrum of superheated steam-treated wheat flour had lower values than native wheat flour, indicating the lower short-range order of starch molecules. This result could be due to the destruction of hydrogen bonds during superheated steam treatment, which uncoiled the double helices in starch molecules [47].

### 3.3. Chemical Composition Measurement of Spelt Flour

**Figure 5:**
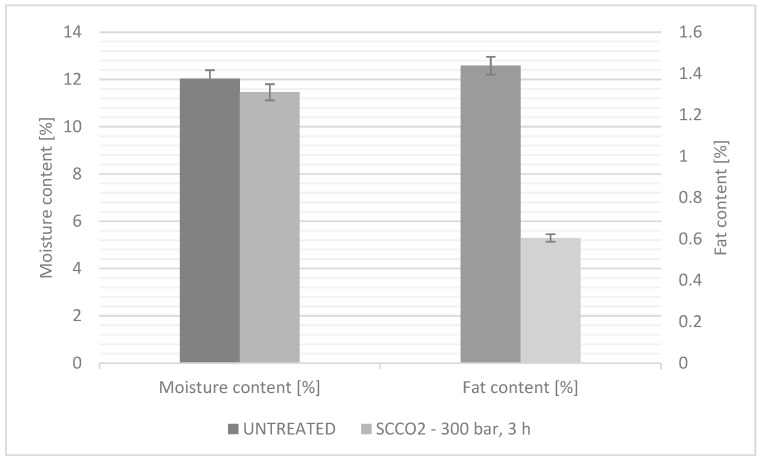
The moisture and fat content [%] comparison of untreated and scCO_2_-treated spelt flour.

Chemical compositions may affect the flour properties of dough kneading (water absorption rate), gluten network formation, dough properties (hardness, viscosity, elasticity, extensibility, plasticity, water retention, etc.) and cooking characteristics (shape retention, chewing viscosity, hardness, shrinkage, etc.), which are especially important in bakery production [48]. The results in Figure 5 show that the spelt flour contains a relatively high fat content (1.4%), whereby by exposing spelt flour in scCO_2_ medium, it was demonstrated that the fat content was reduced to 0.6%, which is 58% less fat in our scCO_2_-treated spelt flour sample. The lower fat content of flour also has some permeability, as this reduces the possibility of flour rancidity and, also, from a dietary point of view, flour is deficient in calories and, consequently, healthier.

The moisture content of the flour is important since the higher moisture content causes a lower amount of dry solids in the flour. Additionally, moisture values inform us indirectly about flour storage conditions. Flour specifications usually limit the flour moisture to 14% or less because flour with greater than 14% moisture is not stable at room temperature. Excessive moisture facilitates mould growth [49], since organisms naturally present in the flour will start to grow at high moistures, producing off odors and flavors. It has been shown that the use of scCO_2_ technology has also reduced the fat content of spelt flour, which consequently reduced the possibility of flour rancidity. An excellent result is also that the flour maintained nutritional value and sensory quality after scCO_2_ treatment. That is undoubtedly an important factor influencing the durability of flour. 

**Figure 6:**
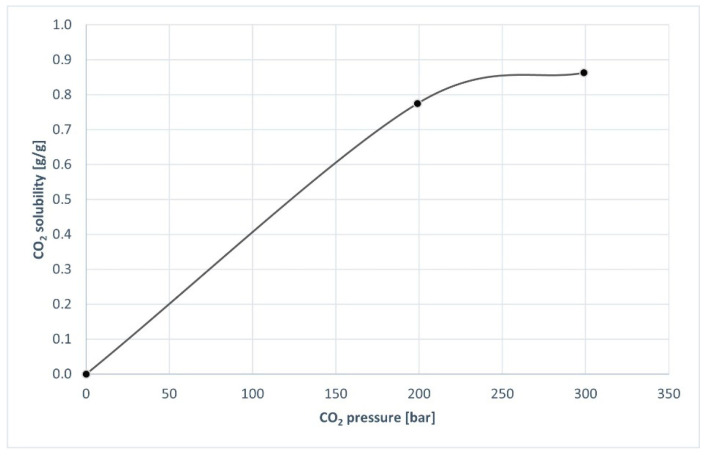
The solubility of CO_2_ in spelt flour at 35 °C.

Microbial growth on foods is inhibited with CO_2_ used at an adequately high concentration. The freshness of foods and their shelf life is therefore extended. Moreover, the high solubility of CO_2_ in foods enables antimicrobial effect, whereby CO_2_ gas is readily soluble in aqueous and fatty foods and, at lower temperature, a higher level of solubility occures. Investigating the effect of CO_2_ solubility on spelt flour, the amount of CO_2_ (or measured solubility of CO_2_) in spelt flour was measured using MSB at a temperature of 35 °C. CO_2_ solubility was determined under the same conditions as scCO_2_ treatment of spelt flour in a high-pressure batch reactor to determine the connection between CO_2_ solubility and enzyme activity in spelt flour. As seen in Figure 6, CO_2_ solubility increased as saturation pressure increased, indicating that CO_2_ gas dissolves, resulting in a sorption behaviour that follows Henry’s law [44]. For the case of spelt flour, CO_2_ solubility of 0.77 g/g was determined at 200 bar and 35 °C, and 0.86 g/g at 300 bar and 35 °C. It has been reported that the higher level of CO_2_ solubility at lower temperatures favours chilled storage to allow a reasonable level of CO_2_ accumulation during the required shelf life of the product [50].

### 3.4. Breadmaking

**Figure 7:**
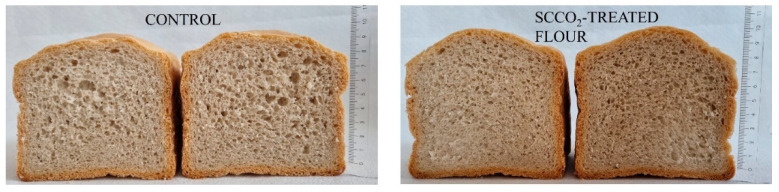
Effect of scCO_2_-treated spelt flour on breadmaking.

In many studies it was found out that enzymes application leads to the improved sensory quality of bread, which is the consequence of the improved flour quality. In addition, a dough that has more machine tolerance was created, and bread structure was enhanced [51]. The focus of this study was investigating the effects of change in enzyme activity in spelt flour by scCO_2_ treatment on flour properties and the quality of baked bread. Figure 7 shows the difference in the final product—bread with untreated and scCO_2_-treated spelt flour. It can be seen that bread made from scCO_2_-treated spelt flour was higher and, consequently, had a higher specific surface volume, which indicated that scCO_2_ has a positive effect on exposed spelt flour, where inactivation of certain enzymes and hyperactivation of α-amylase enzyme gave bread with better characteristics compared to control bread, where untreated spelt flour was used. Additionally, our findings from the determination of the activity of individual enzymes are consistent with the final product, as the presence of the enzyme α-amylase had a positive effect on the rising process, whereby it improved the internal texture of bread and increased the volume of bread. The average height of bread calculated using the ImageJ program for bread baked from untreated (control) spelt flour in three different samples was 10.2 ± 0.25 cm, while the average height of bread beaked from scCO_2_-treated spelt flour in three different samples was 10.4 ± 0.17 cm. In accordance with these measurements, measurements of the specific surface volume of the bread were also carried out, and it was found that the bread from untreated spelt flour has a specific surface volume 2.74 ± 0.1 cm^3^/g, which is slightly lower than bread baked from scCO_2_-treated spelt flour with specific volume 2.81 ± 0.08 cm^3^/g.

Usually the quality of the final product changes with different processing techniques of flour or other food products. Our study shows that the quality of flour has improved, which is clear from the final product and its specific properties.

## 4. Conclusions

Consumers are becoming more aware of the benefits of living a healthier lifestyle and maintaining fat-free and other diets. During this study, indirect baking quality parameters were significantly influenced by scCO_2_ treatment of spelt flour. This was reflected in the results of the α-amylase, lipase, POD, PPO, and protease enzyme activity measurements and other characteristic differences in the final product—bread with better properties compared to the control product from flour that was not exposed to scCO_2_ conditions. Fat content in scCO_2_-treated spelt flour significantly decreased due to scCO_2_ exposure, which is essential in reducing the rancidity of flour. In addition, it can be said that, by inactivating unwanted enzymes such as POD and PPO, scCO_2_ treatment affected the prolongation of spelt flour shelf life. In addition, high-pressure processing is more sustainable than conventional processes, which, besides the benefits in terms of non-thermal inactivation of enzymes, and the minimal effects on nutritional and organoleptic properties of food products, is considered to be environmentally friendly. A novelty of this study is the approach in which enzymes are inactivated in the flour using scCO_2_ technology. This study demonstrates that using scCO_2_ technology in flour regulates the activity of individual enzymes in flour. It is extremely important that this process does not negatively affect the quality of flour as a raw material for bakery products. The results of the present study could be used as the basis for the future optimization of spelt flour characteristics and to extend the shelf life, with further studies to be carried out. Considering the obtained results, scCO_2_ exposure of spelt flour can improve some rheological properties of dough and positively affect specific activities of flour enzymes that are important in the production of the final product.

## Data Availability

Data supporting reported results can be received upon request.
